# Evaluating mental health support by healthcare providers for patients with atopic dermatitis: A cross‐sectional survey

**DOI:** 10.1002/ski2.408

**Published:** 2024-06-15

**Authors:** Sheena Chatrath, Allison R. Loiselle, Jessica K. Johnson, Wendy Smith Begolka

**Affiliations:** ^1^ University of Illinois College of Medicine Chicago Illinois USA; ^2^ National Eczema Association Novato California USA

## Abstract

**Importance:**

Atopic dermatitis (AD) is associated with psychosocial symptoms, resulting in significant mental health burden and reduced quality of life.

**Objective:**

To understand mental health support received by patients from their primary eczema provider.

**Design:**

We administered a cross‐sectional survey (*N* = 954) to US caregivers and adult patients with AD.

**Setting:**

The National Eczema Association (United States) conducted an online survey in October 2022 among self‐selected patients and caregivers.

**Outcome:**

Patients and caregivers of AD patients reported on mental health conversations and types of mental health support received by their/their child's provider.

**Results:**

Many patients did not discuss (41.9%) or were not asked (50.5%) about their mental health by their eczema provider, and 64% reported not receiving a mental health referral. Patients were more likely to receive mental health support if they were male (2.00 [1.08–3.69]), low or middle education level (4.89 [2.10–11.36], 2.71 [1.36–5.40]), or had purchased insurance policies (4.43 [1.79–10.98]). Providers were most likely to refer patients to counseling services (22.5%), followed by alternative mental health therapy (14.9%), cognitive behavioural therapy (13.3%) and peer/social support groups (12.2%).

**Conclusion:**

Despite the strong association between AD and mental health conditions, there is a significant proportion of patients that report not receiving mental health support from their/their child's primary eczema provider. Screening with validated measures may improve the identification of patients requiring additional support. Future research should evaluate the efficacy of mental health resources and barriers to accessing and referring patients for mental health care.

**What is already known about this topic?**

- Patients with atopic dermatitis (AD) have high levels of mental health burden throughout the course of the disease.

**What does this study add?**

- There is a significant proportion of patients with AD that do not receive adequate mental health support from their primary eczema provider despite experiencing significant mental health symptoms.
- Validated screening measures may improve the identification of patients who require or desire additional mental health support.

## 1 INTRODUCTION

Atopic dermatitis (AD), commonly known as eczema, is a chronic inflammatory skin disorder associated with several psychosocial impacts including anxiety, depression, social isolation, financial and occupational burden, and decreased quality of life.^1^, ^2^, ^3^, ^4^, ^5^ In comparison to the general population, patients with AD are twice as likely to develop anxiety and depression,^6^ and have an increased risk of suicidal ideation.^7^, ^8^ Moreover, previous studies have shown an association between the severity of AD and mental health symptoms,^9^, ^10^ with symptoms appearing as early as 4 years of age, and progressing as the patient ages.^11^

Despite the association between mental health disorders and AD, there is poor utilization of mental health resources among patients with eczema.^12^, ^13^ In a survey of 1118 adult and pediatric caregiver respondents, 85.6% reported no out of pocket expense for mental health specialists or counseling services.^12^ Additionally, in a cross‐sectional study of pediatric patients with AD, only 53% of patients with severe mental health distress and 13% overall, utilized mental health resources.^13^

While several factors may contribute to the disconnect between the utilization of mental health resources and the mental health burden associated with AD,^14^ there is no known literature about the evaluation and referral practices for mental health symptoms by AD providers. The purpose of this study is to understand and elucidate the mental health support that patients with AD receive from their primary eczema provider.

## 2 METHODS

A cross‐sectional survey was administered using Qualtrics^15^ between October‐November 2022 through the National Eczema Association (NEA) in the United States to primary caregivers of pediatric patients (8–17 years) and adults (≥18 years) with a self‐reported diagnosis of AD. Participants were recruited through the NEA website, email, social media, and the EczemaWise smartphone application. Participants were eligible for the survey if they were U.S. residents and excluded if they were unable to complete the questionnaire. The survey was designed in partnership with patient volunteers that provided feedback on content and literacy level. Prior to completing the online survey, respondents provided electronic informed consent. No personally identifiable data was collected, and individual results of the survey were only accessed by the NEA. Participants of this survey were entered to receive 1 of 15 amazon gift cards. Duplicate/bot responses were screened out using Qualtrics' built in mechanisms as well as CAPTCHA and hidden questions. This study was reviewed by the Western Institutional Review Board Copernicus Group (WCG IRB) and was identified as exempt.

### 2.1 Outcome measures

The self‐reported survey measures examined the caregiver and patient experience addressing mental health concerns with their regular eczema provider. The questionnaire included sociodemographic factors, mental health evaluation, and referral to mental health services. Mental health symptoms were screened using the PHQ‐9 questionnaire (9 questions, 0–3 per question, score of 0–27).^16^, ^17^ Mental health services were defined as follows: ‘Mental health services may include, but are not limited to, counseling with a mental health provider, cognitive behavioural therapy (CBT), social support groups, alternative mental health therapy (e.g. music/art therapy), medication.’

### 2.2 Statistical analysis

All statistical analyses were conducted using SAS 9.4 (SAS Institute). A *p* value of <0.05 was used to indicate statistical significance. Baseline statistics were estimated to characterize the population. Multivariate logistic regression models and logistic regression models were constructed to analyze the outcome variables. Dependent variables included time from AD diagnosis until speaking with an eczema provider about mental health, whether the primary eczema provider asked about mental health symptoms at any visit, and whether the patient/caregiver received a referral for mental health services. Predictors included age, gender, race, ethnicity, income, geographic region, education, insurance type, and primary eczema provider type. Adjusted odds ratios (aOR) and adjusted 95% CI were estimated for all models.

## 3 RESULTS

### 3.1 Population characteristics

In total, 1496 people took the survey, 991 of whom met inclusion criteria. An additional 37 responses were removed for those who finished fewer than 50% of the survey questions. Therefore, analysis was conducted on 954 respondents (954/991; completion rate 96.3%) The majority of respondents were adult patients (83.3%), female (68.1%), White (67.0%), from an urban geographic area (89.6%), and saw a specialist (allergist or dermatologist) as their primary eczema provider (64.6%) (Table 1).

**TABLE 1:** Characteristics of the study population (*N* = 954).

Connection to eczema % (*n*)	
Adult patient	83.3% (795)
Caregiver of pediatric patient	16.7% (159)
Age (mean + −SD)	
Adult patient	38.0 + −17.0
Caregiver	37.8 + −8.1
Child (as reported by caregiver)	11.4 + −2.8
Primary respondent % (*n*)	
Female gender	68.1% (650)
Male gender	29.8% (284)
Other gender/prefer not to answer	2.1% (20)
Adult patient	83% (795)
Child patient	17% (159)
Respondent race % (*n*)	
White	67.0% (639)
Black or African American	9.3% (89)
Asian or Asian American	9.6% (92)
Multiracial/other	14.2% (136)
Respondent ethnicity % (*n*)	
Hispanic	25.3% (242)
Non‐Hispanic	75.0% (715)
Annual income % (*n*)	
Low income (≤$49K)	33.4% (319)
Middle income ($50–149 K)	55.7% (531)
High income (≥150K)	10.9% (104)
RUCA % (*n*)	
Urban	89.6% (835)
Large rural city/town	4.2% (39)
Small and isolated small rural town	6.2% (58)
AD severity % (*n*)	
Clear/almost clear	22.3% (213)
Mild	28.4% (271)
Moderate	31.4% (300)
Severe	17.7% (169)
Age AD diagnosis % (*n*)	
Under 2 years of age	24.5% (234)
2–5 years	17.8% (170)
6–9 years	11.8% (113)
10–17 years	17.7% (169)
18 years or older	25.4% (242)
Insurance type % (*n*)	
Employer‐sponsored coverage	39.0% (372)
Medicaid or state assistance	13.5% (129)
Medicare/tricare/VA benefit	23.5% (224)
Commercial market/state/federal	17.4% (166)
Other insurance	3.5% (33)
No insurance	3.1% (30)
Primary provider type % (*n*)	
Specialist (dermatology/allergy)	64.6% (616)
Non‐specialist*	24.7% (236)
No provider	10.7% (102)
Mental health symptoms in the past month % (*n*)	
0 days	12.7% (121)
1–5 days	36.5% (348)
6–10 days	24.7% (236)
11–15 days	11.5% (110)
16+ days	9.9% (94)
Every day	4.7% (45)
Respondent education % (*n*)	
Limited*	15.3% (146)
Average*	33.2% (317)
Advanced*	51.5% (491)

*Note*: *Non‐specialist (Family/General Practitioner, Internal Medicine Physician, Pediatrician, PA/Nurse Practitioner, Alternative Medicine Doctor/Other); *Limited (Less than high school/Completed some high school/High school graduate); Average *(Technical post‐secondary degree/Completed some college); *Advanced (Four‐year college degree/Masters degree/Doctorate).

Abbreviations: AD, atopic dermatitis; RUCA, rural‐urban commuting area; VA, veterans affairs.

### 3.2 Mental health evaluation by primary eczema provider

Over 40% of respondents (41.9%) never spoke about mental health with their primary eczema provider, 50.5% were never asked about mental health during any visit, and 64.1% of respondents did not receive a referral for mental health services by their provider (Table 2). Of those receiving a referral, 56.6% of respondents utilized the recommended mental health services.

**TABLE 2:** Dependent variables and frequency distribution.

	*N* = 954
Time from AD diagnosis until speaking with primary eczema provider about mental health % (*n*)	
Less than 1 month	10.5% (100)
1–6 months	24.3% (232)
7–12 months	9.9% (94)
More than 1 year	13.4% (128)
Never spoke about mental health	41.9% (400)
Did the provider ask about mental health during ANY visit?	
No	39.4% (376)
No, but wanted to be asked	11.1% (106)
Yes, but did not want to be asked	16.8% (160)
Yes	32.7% (312)
Did the provider direct you/your child to mental health resources or services?	
No	64.1% (611)
Yes	36.0% (343)

Abbreviation: AD, atopic dermatitis.

Male patients, patients with limited (≤ high school), or average (>high school to some college) education, and those with purchased insurance policies, were more likely to talk about mental health 7–12 months after their AD diagnosis (aOR [95%CI]: 2.00 [1.08–3.69], 4.89 [2.10–11.36], 2.71 [1.36–5.40], 4.43 [1.79–10.98]). Patients with low income, 35–64 years of age, and those without a provider, were less likely to have ever discussed mental health with their primary eczema provider (1.98 [1.16–3.39], 2.48 [1.18–5.23], 2.59 [1.12–6.00]). Patients whose primary eczema provider was not a specialist were more likely to be asked about mental health compared to patients primarily seeing a specialist (2.58 [1.78–3.74]) (Table 3, Table S1).

**TABLE 3:** Multivariate logistic regression models.

Predictors	1–6 months	7–12 months	More than a year	Never
	OR (95% CI) *p*‐value	OR (95% CI) *p*‐value	OR (95% CI) *p*‐value	OR (95% CI) *p*‐value
Time from AD diagnosis to speaking with HCP about mental health				
Age				
0–17 (ref)	–	–	–	–
35–64	0.48 (0.22–1.02) 0.0562	0.77 (0.31–1.92) 0.5769	1.12 (0.49–2.57) 0.7963	2.48 (1.18–5.23) 0.0171
65+	0.22 (0.08–0.63) 0.0047	0.30 (0.07–1.27) 0.1015	0.72 (0.24–2.10) 0.5426	1.87 (0.78–4.49) 0.164
Gender				
Female (ref)	–	–	–	–
Male	1.68 (1.02–2.76) 0.043	2.00 (1.08–3.69) 0.0269	0.87 (0.49–1.54) 0.6216	0.47 (0.29–0.77) 0.003
Income				
Middle (ref)	–	–	–	–
Low	1.40 (0.79–2.48) 0.2518	1.09 (0.54–2.20) 0.8104	2.53 (1.36–4.70) 0.0033	1.98 (1.16–3.39) 0.0127
RUCA				
Urban (ref)	–	–	–	–
Large rural city town	1.18 (0.82–1.70) 0.3715	1.41 (0.92–2.16) 0.1146	0.53 (0.33–0.84) 0.0074	1.0 (0.71–1.41) 0.9841
Small/isolated rural town	1.32 (1.01–1.74) 0.0463	1.36 (0.99–1.89) 0.0618	0.59 (0.42–0.84) 0.0030	0.39 (0.29–0.52) <0.0001
Education				
Advanced (ref)	–	–	–	–
Limited	2.56 (1.24–5.28) 0.0112	4.89 (2.10–11.36) 0.0002	0.90 (0.40–2.02) 0.7914	0.59 (0.29–1.20) 0.1461
Average	2.42 (1.41–4.14) 0.0013	2.71 (1.36–5.40) 0.0047	0.63 (0.34–1.17) 0.1456	0.74 (0.45–1.22) 0.2391
Insurance type				
Employer–sponsored (ref)	–	–	–	–
Medicaid or state assistance	1.85 (0.88–3.91) 0.1072	1.91 (0.77–4.73) 0.1604	0.72 (0.32–1.60) 0.4153	0.49 (0.24–0.98) 0.0451
Medicare/tricare or VA benefit	1.52 (0.82–2.83) 0.1848	1.82 (0.83–3.99) 0.1342	0.58 (0.30–1.1) 0.1162	0.47 (0.27–0.83) 0.0086
Purchased policy	5.02 (2.32–10.88) <0.0001	4.43 (1.79–10.98) 0.0013	0.61 (0.23–1.58) 0.308	0.88 (0.41–1.89) 0.7415
Primary eczema provider type				
Specialist (ref)	–	–	–	–
Non‐specialist	0.83 (0.48–1.41) 0.4824	1.30 (0.69–2.45) 0.4202	0.93 (0.51–1.68) 0.8091	0.55 (0.32–0.92) 0.0227
No provider	0.42 (0.14–1.24) 0.1157	0.41 (0.10–1.71) 0.2201	0.76 (0.26–2.25) 0.6193	2.59 (1.12–6.00) 0.0261

Predictors	No, but wanted to be asked	Yes	Yes, but did not want to be asked
	OR (95% CI) *p*‐value	OR (95% CI) *p*‐value	OR (95% CI) *p*‐value
Primary HCP asked about mental health at any AD visit			
Age			
0–17 (ref)	–	–	–
18–34	5.52 (1.88–16.24) 0.0019	1.25 (0.79–1.97) 0.3446	1.38 (0.82–2.35) 0.2277
35–64	2.60 (0.86–7.93) 0.0922	0.46 (0.28–0.76) 0.0021	0.19 (0.09–0.38) <0.0001
65+	2.69 (0.76–9.52) 0.1239	0.26 (0.13–0.52) 0.0001	0.06 (0.01–0.28) 0.0003
Gender			
Female (ref)	–	–	–
Male	0.57 (0.30–1.07) 0.0801	2.81 (1.98–3.99) <0.0001	2.60 (1.71–3.95) <0.0001
Race			
White (ref)	–	–	–
Asian or Asian American	3.19 (1.65–6.16) 0.0005	0.55 (0.29–1.03) 0.0624	0.76 (0.37–1.55) 0.4461
Multiracial/other	1.85 (1.01–3.41) 0.0477	0.76 (0.47–1.23) 0.2615	0.85 (0.47–1.55) 0.6043
Ethnicity			
Non–Hispanic (ref)	–	–	–
Hispanic	1.03 (0.58–1.83) 0.9225	1.83 (1.25–2.68) 0.0019	2.47 (1.58–3.86) <0.0001
Income			
Middle (ref)	–	–	–
High	0.80 (0.39–1.62) 0.531	0.56 (0.34–0.95) 0.0305	0.17 (0.06–0.44) 0.0003
RUCA			
Urban (ref)	–	–	–
Large rural city town	0.59 (0.40–0.88) 0.0088	1.27 (1.01–1.61) 0.0428	0.96 (0.71–1.30) 0.7859
Small/isolated rural town	<0.001 (<0.001–>999.9) 0.9105	1.78 (1.46–2.17) <0.0001	1.89 (1.50–2.38) <0.0001
Education			
Advanced (ref)	–	–	–
Limited	0.59 (0.28–1.28) 0.1829	1.64 (1.03–2.63) 0.0392	3.81 (2.13–6.82) <0.0001
Average	0.62 (0.37–1.07) 0.0845	1.33 (0.93–1.90) 0.1165	3.46 (2.20–5.45) <0.0001
Insurance type			
Employer–sponsored (ref)	–	–	–
Medicaid or state assistance	1.28 (0.62–2.65) 0.5062	2.76 (1.65–4.63) 0.0001	4.48 (2.32–8.66) <0.0001
Medicare/tricare or VA benefit	0.67 (0.34–1.30) 0.2357	2.12 (1.40–3.23) 0.0004	2.97 (1.70–5.20) 0.0001
Purchased policy	0.38 (0.14–1.01) 0.0522	2.35 (1.45–3.80) 0.0006	5.74 (3.24–10.18) <0.0001
Other insurance	1.27 (0.68–2.36) 0.4577	0.45 (0.25–0.81) 0.0078	0.30 (0.13–0.70) 0.005
Provider type			
Specialist (ref)	–	–	–
Non–specialist	1.03 (0.56–1.89) 0.9275	2.58 (1.78–3.74) <0.0001	1.45 (0.90–2.34) 0.123
No provider	1.27 (0.68–2.36) 0.4577	0.45 (0.25–0.81) 0.0078	0.30 (0.13–0.70) 0.005

Abbreviations: AD, atopic dermatitis; HCP, health care provider; RUCA, rural‐urban commuting area; VA, veterans affairs.

### 3.3 Patient preference of mental health evaluations

Adults 18–34 and 35–64, Asian/Asian American patients, and Multiracial patients, were less likely to be asked about their mental health at any visit but wanted to be asked (5.52 [1.88–16.24], 2.60 [0.86–7.93], 3.19 [1.65–6.16], 1.85 [1.01–3.41]). Male patients (2.81 [1.98–3.99]), Hispanic ethnicity (1.83 [1.25–2.68]), limited education (1.64 [1.03–2.63]), small, rural geographic area (1.78 [1.46–2.17]), Medicaid/State assistance (2.76 [1.65–4.63]), Medicare/Tricare/veterans affairs (VA) Benefit (2.12 [1.40–3.23]), or purchased insurance policies (2.35 [1.45–3.80]) were more likely to be asked about mental health, but also did not want to be asked (2.60 [1.71–3.95], 2.47 [1.58–3.86], 3.81 [2.13–6.82], 4.48 [2.32–8.66], 2.97 [1.70–5.20], 5.74 [3.24–10.18]) (Table 3, Table S1).

### 3.4 Mental health referral by primary eczema provider

**FIGURE 1:**
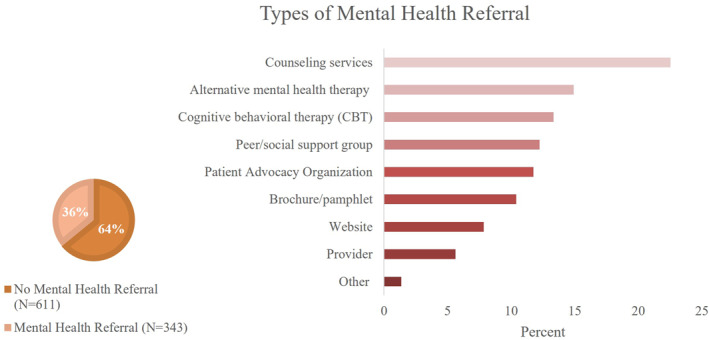
Types of mental health referrals by providers.

Similarly to those that were asked about mental health, respondents were more likely to receive a referral for mental health services if they were male (2.58 [1.90–3.49]), of Hispanic ethnicity (2.75 [1.99–3.81]), from a small/rural town (2.19 [1.84–2.60]), had limited or average education completion (4.74 [3.11–7.24], 2.48 [1.79–3.42]), had Medicaid/State assistance (3.07 [1.96–4.81]), Medicare/Tricare/VA Benefit (2.66 [1.81–3.92]), purchased policy insurance (2.91 [1.93–4.93]), or saw a non‐specialist as their primary eczema provider (1.80 [1.30–2.48]) (Table 4, Table S2). Of the patients receiving a mental health referral, the most common services included counseling (22.5%), alternative mental health therapy (14.9%), CBT (13.3%), and peer/social support groups (12.2%) (Figure 1).

**TABLE 4:** Logistic regression model.

Predictors	Yes
	OR (95% CI) *p*‐value
HCP referred to mental health resources	
Age	
0–17 (ref)	–
35–64	0.28 (0.18–0.45) <0.0001
65+	0.09 (0.04–0.22) <0.0001
Gender	
Female (ref)	–
Male	2.58 (1.90–3.49) <0.0001
Race	
White (ref)	–
Asian or Asian American	0.59 (0.35–1.00) 0.0493
Ethnicity	
Non‐Hispanic (ref)	–
Hispanic	2.75 (1.99–3.81) <0.0001
Income	
Middle (ref)	–
High	0.36 (0.20–0.62) 0.0003
RUCA	
Urban (ref)	–
Small/isolated rural town	2.19 (1.84–2.60) <0.0001
Education	
Advanced (ref)	–
Limited	4.74 (3.11–7.24) <0.0001
Average	2.48 (1.79–3.42) <0.0001
Insurance type	
Employer‐sponsored (ref)	–
Medicaid or state assistance	3.07 (1.96–4.81) <0.0001
Medicare/tricare/VA	2.66 (1.81–3.92) <0.0001
Purchased policy	2.91 (1.93–4.39) <0.0001
Primary eczema provider type	
Specialist (ref)	–
Non‐specialist	1.80 (1.30–2.48) 0.0003
No provider	0.28 (0.15–0.53) <0.0001

Abbreviations: HCP, health care provider; RUCA, rural‐urban commuting area; VA, veterans affairs.

## 4 DISCUSSION

This is the first study to comprehensively explore the mental health support received by patients with AD from their primary eczema provider. In this study, we found a significant portion of patients were not asked or did not speak to their AD provider about mental health, and most patients did not receive a referral for mental health support. Previous literature has demonstrated similar rates of mental health screening in dermatology.^18^, ^19^, ^20^ One survey of 9435 AD visits found that only 1.2% of patients were screened for depression with similar screening rates among both dermatologists and non‐dermatologists.^18^ In contrast to this study, we found mental health concerns were addressed more frequently among non‐specialist physicians than specialists. This discrepancy may be due to the recently updated United States Preventive Services Task Force guidelines on screening for anxiety and depression,^21^, ^22^, ^23^, ^24^, ^25^ the training primary care physicians receive in addressing mental health, and the frequency of patients seen for anxiety and depression.^26^ Moreover, in a survey of 56 dermatology residents, 64% reported mental health screening was not included in their training curriculum, and 45% reported discomfort with addressing mental health concerns due to lack of training.^20^ Future research should evaluate whether increased training on mental health screening and support improves the comfort level and management of mental health conditions by AD providers.

In addition to the overall rates of mental health support, we found patients were more likely to be asked about mental health if they were male, had lower education levels, or had purchased insurance policies. Conversely, adult patients, those with low income, and patients of Asian or Multiracial ethnicity were less likely to be asked. Interestingly, multiple demographics including male patients and those with lower education levels, did not want to be asked about their/their child's mental health, whereas Asian, Multiracial, and adult patients wanted to be asked. These findings suggest that mental health support may be offered based on perceived need rather than validated screening measures. Moreover, patients with mental health symptoms may be hesitant to discuss their mental health concerns due to ongoing stigma towards mental health conditions or distrust towards their healthcare provider.^27^, ^28^ In fact, previous literature has shown that patients report fear of stigmatization, dismissed concerns, and failure to incorporate treatment preferences by their provider as barriers to seeking mental health support.^29^ While reducing mental health stigma is an ongoing process, providers may improve mental health outcomes through normalizing mental health discussions as well as incorporating holistic, patient‐centred care and shared decision making during patient visits.^29^, ^30^

Males, patients of Hispanic ethnicity, those with lower education, public or purchased insurance, and those with a non‐specialist healthcare provider, were more likely to receive a referral for mental health services. Additionally, most patients were referred to counseling services, with fewer patients receiving a referral for CBT or other mental health services. The wide range of mental health referrals may reflect limited access or barriers to mental health resources in the providers' geographic region, as well as a lack of research regarding which mental health interventions are most effective for patients with AD. Several previous studies have illustrated an unmet need for psychiatric interventions.^31^, ^32^, ^33^, ^34^ In a cross‐sectional study of 2800 psychiatrists, over one third reported an inability to provide enough psychiatric outpatient appointments, affecting approximately 25% of their patient population.^34^ In another survey of 2194 adults, patients reported delays in receiving care due to provider‐related barriers (language, culture, and trustworthiness), family/work barriers, and barriers to accessing care.^35^ These findings suggest that in addition to provider support, community and national resources such as patient/peer support groups and patient advocacy organizations may be helpful in decreasing the overall mental health burden. Future research should evaluate the barriers to mental health care and the efficacy of mental health interventions for patients with AD.

Strengths of this study include a large sample size including patients and caregivers across the United States as well as the patient and caregiver perspective regarding mental health support received from their primary eczema provider. Limitations to this study include a retrospective, self‐reported survey without physician confirmation of AD, caregiver proxy reporting on the mental health of their child as well as a lack of insight from healthcare providers regarding their mental health screening and referral behaviours.

## 5 CONCLUSION

A significant portion of adult AD patients and caregivers of pediatric AD patients report not receiving mental health support from their/their child's primary eczema provider. Additionally, many patients who did receive support, preferred not to be asked about their/their child's mental health. Conversely, those who did not receive mental health support, preferred to have been asked about their/their child's mental well‐being. Screening for mental health symptoms with a validated approach and/or questionnaire prior to a medical encounter may help identify AD patients that need and desire additional support. Future research is needed to understand the barriers to utilizing mental health resources in dermatologic practice as well as the efficacy of those resources.

## CONFLICT OF INTEREST STATEMENT

This study was sponsored by the National Eczema Association (NEA). **JKJ**, **AL**, and **WSB** are employees of the NEA. **WSB** received advisory board honoraria from Pfizer and Incyte, and research grants from Pfizer.

## AUTHOR CONTRIBUTIONS

**Sheena Chatrath**: Formal analysis (lead); methodology (lead); project administration (lead); writing – original draft (lead); writing – review & editing (lead). **Allison R. Loiselle**: Formal analysis (supporting); writing – review & editing (supporting). **Jessica K. Johnson**: Writing – review & editing (supporting). **Wendy Smith Begolka**: Conceptualisation (lead); supervision (lead); writing – review & editing (supporting).

## ETHICS STATEMENT

Not applicable.

## PATIENT CONSENT

Not applicable.

## Supporting information

Supporting Information S1

## Data Availability

The data underlying this article will be shared on reasonable request to the corresponding author.
