# Supplementary material for: Evaluating mental health support by healthcare providers for patients with atopic dermatitis: A cross‐sectional survey
Source: Skin Health Dis. 2024 Jun 15;4(5):e408. doi: 10.1002/ski2.408 (PMC11442069; doi:10.1002/ski2.408)
Supplement: Supplementary file 1 — Supporting Information S1 [file SKI2-4-e408-s001.docx]

Supplemental Table 1: Multivariate Multiple Logistic Regression Models

| **Time from AD diagnosis to speaking with HCP about mental health** | | | | |
| --- | --- | --- | --- | --- |
| **Predictors** | 1-6 months  **OR (95% CI) P-value** | 7-12 months  **OR (95% CI) P-value** | More than a year  **OR (95% CI) P-value** | Never  **OR (95% CI) P-value** |
| Age    0-17 (ref)  18-34  35-64  65+ | –  0.80 (0.42-1.54) 0.5029  0.48 (0.22-1.02) 0.0562  **0.22 (0.08-0.63) 0.0047** | –  0.85 (0.39-1.85) 0.6746  0.77 (0.31-1.92) 0.5769  0.30 (0.07-1.27) 0.1015 | –  0.92 (0.43-1.95) 0.823  1.12 (0.49-2.57) 0.7963  0.72 (0.24-2.10) 0.5426 | –  1.69 (0.85-3.36) 0.1366  **2.48 (1.18-5.23) 0.0171**  1.87 (0.78-4.49) 0.164 |
| Gender  Female (ref)  Male  Other | –  **1.68 (1.02-2.76) 0.043**  2.00 (0.22-18.63) 0.5419 | –  **2.00 (1.08-3.69) 0.0269**  1.76 (0.15-21.05) 0.6559 | –  0.87 (0.49-1.54) 0.6216  0.48 (0.03-7.97) 0.6067 | –  **0.47 (0.29-0.77) 0.003**  0.48 (0.03-7.97) 0.6067 |
| Race  White (ref)  Asian/Asian  American  Black/African  American  Multiracial/Other | –  1.68 (0.54-5.26) 0.3704  0.78 (0.37-1.66) 0.5181  0.87 (0.45-1.69) 0.6803 | –  1.81 (0.52-6.24) 0.3496  0.44 (0.15-1.27) 0.1282  0.31 (0.11-0.85) 0.0232 | –  2.74 (0.85-8.82) 0.0908  1.07 (0.47-2.43) 0.8715  0.69 (0.32-1.49) 0.3476 | –  2.54 (0.86-7.50) 0.0909  0.63 (0.31-1.29) 0.2085  0.82 (0.44-1.52) 0.5284 |
| Ethnicity  Non-Hispanic (ref)  Hispanic | –  1.63 (0.95-2.79) 0.0739 | –  1.72 (0.90-3.27) 0.0989 | –  0.63 (0.33-1.19) 0.1552 | –  0.62 (0.36-1.06) 0.0807 |
| Income  Middle (ref)  Low    High | –  1.40 (0.79-2.48) 0.2518  0.57 (0.24-1.34) 0.1985 | –  1.09 (0.54-2.20) 0.8104  0.77 (0.27-2.20) 0.6314 | –  **2.53 (1.36-4.70) 0.0033**  1.98 (0.84-4.65) 0.1175 | –  **1.98 (1.16-3.39) 0.0127**  1.45 (0.70-3.00) 0.3202 |
| RUCA  Urban (ref)  Large Rural  City Town  Small/Isolated  Rural Town | –  1.18 (0.82-1.70)  0.3715  1.32 (1.01-1.74)  0.0463 | –  1.41 (0.92-2.16)  0.1146  1.36 (0.99-1.89)  0.0618 | –  **0.53 (0.33-0.84)**  **0.0074**  **0.59 (0.42-0.84)**  **0.0030** | –  1.0 (0.71-1.41)  0.9841  **0.39 (0.29-0.52)**  **<.0001** |
| Education  Advanced (ref)  Limited  Average | –  **2.56 (1.24-5.28) 0.0112**  **2.42 (1.41-4.14) 0.0013** | –  **4.89 (2.10-11.36) 0.0002**  **2.71 (1.36-5.40) 0.0047** | –  0.90 (0.40-2.02) 0.7914  0.63 (0.34-1.17) 0.1456 | –  0.59 (0.29-1.20) 0.1461  0.74 (0.45-1.22) 0.2391 |
| Insurance Type  Employer-sponsored  Coverage (ref)  Medicaid or State  Assistance  Medicare/Tricare  or VA Benefit  Purchased policy    Other Insurance    No Insurance | –  1.85 (0.88-3.91) 0.1072  1.52 (0.82-2.83) 0.1848  **5.02 (2.32-10.88) <.0001**  2.22 (0.41-11.95) 0.3528  4.72 (0.51-43.67) 0.1721 | –  1.91 (0.77-4.73) 0.1604  1.82 (0.83-3.99) 0.1342  **4.43 (1.79-10.98) 0.0013**  1.79 (0.22-14.38) 0.5864  1.76 (0.10-31.32) 0.6996 | –  0.72 (0.32-1.60) 0.4153  0.58 (0.30-1.1)  0.1162  0.61 (0.23-1.58) 0.308  1.45 (0.26-8.11) 0.673  1.18 (0.10-13.91) 0.8949 | –  **0.49 (0.24-0.98) 0.0451**  **0.47 (0.27-0.83)**  **0.0086**  0.88 (0.41-1.89) 0.7415  1.67 (0.36-7.80) 0.5151  3.75 (0.47-29.81) 0.2122 |
| Primary Eczema Provider Type  Specialist (ref)  Non-specialist  No Provider | –  0.83 (0.48-1.41) 0.4824  0.42 (0.14-1.24) 0.1157 | –  1.30 (0.69-2.45) 0.4202  0.41 (0.10-1.71) 0.2201 | –  0.93 (0.51-1.68) 0.8091  0.76 (0.26-2.25) 0.6193 | –  **0.55 (0.32-0.92) 0.0227**  **2.59 (1.12-6.00) 0.0261** |
| **Primary HCP asked about mental health at any AD visit** | | | | |
| **Predictors** | No, but wanted to be asked  **OR (95% CI) P-value** | Yes  **OR (95% CI) P-value** | Yes, but did not want to be asked  **OR (95% CI) P-value** |  |
| Age  0-17 (ref)  18-34  35-64    65+ | –  **5.52 (1.88-16.24) 0.0019**  **2.60 (0.86-7.93) 0.0922**  2.69 (0.76-9.52) 0.1239 | –  1.25 (0.79-1.97) 0.3446  **0.46 (0.28-0.76) 0.0021**  **0.26 (0.13-0.52) 0.0001** | –  1.38 (0.82-2.35) 0.2277  **0.19 (0.09-0.38) <.0001**  **0.06 (0.01-0.28) 0.0003** |  |
| Gender  Female (ref)  Male  Other | –  0.57 (0.30-1.07) 0.0801  4.06 (1.16-14.20) 0.0281 | –  **2.81 (1.98-3.99) <.0001**  1.84 (0.49-6.86) 0.3656 | –  **2.60 (1.71-3.95) <.0001**  1.44 (0.26-8.10) 0.6795 |  |
| Race  White (ref)  Asian or Asian  American  Black or African  American  Multiracial/Other | –  **3.19 (1.65-6.16) 0.0005**  1.97 (0.90-4.31) 0.0892  **1.85 (1.01-3.41) 0.0477** | –  0.55 (0.29-1.03) 0.0624  1.39 (0.82-2.36) 0.2262  0.76 (0.47-1.23) 0.2615 | –  0.76 (0.37-1.55) 0.4461  0.74 (0.35-1.56) 0.4284  0.85 (0.47-1.55) 0.6043 |  |
| Ethnicity  Non-Hispanic (ref)    Hispanic | –  1.03 (0.58-1.83) 0.9225 | –  **1.83 (1.25-2.68) 0.0019** | –  **2.47 (1.58-3.86) <.0001** | – |
| Income  Middle (ref)  Low    High | –  0.91 (0.55-1.50) 0.7161  0.80 (0.39-1.62) 0.531 | –  0.78 (0.55-1.11) 0.1663  **0.56 (0.34-0.95) 0.0305** | –  0.70 (0.45-1.08) 0.1081  **0.17 (0.06-0.44) 0.0003** |  |
| RUCA  Urban (ref)  Large Rural  City Town  Small/Isolated  Rural Town | –  **0.59 (0.40-0.88)**  **0.0088**  <0.001(<0.001->999.9)  0.9105 | –  **1.27 (1.01-1.61)**  **0.0428**  **1.78 (1.46-2.17)**  **<.0001** | –  0.96 (0.71-1.30)  0.7859  **1.89 (1.50-2.38)**  **<.0001** |  |
| Education  Advanced (ref)    Limited  Average | –  0.59 (0.28-1.28) 0.1829  0.62 (0.37-1.07) 0.0845 | –  **1.64 (1.03-2.63) 0.0392**  1.33 (0.93-1.90) 0.1165 | –  **3.81 (2.13-6.82) <.0001**  **3.46 (2.20-5.45) <.0001** |  |
| Insurance Type    Employer-sponsored  Coverage (ref)  Medicaid or State  Assistance  Medicare/Tricare  or VA Benefit  Purchased Policy  Other Insurance  No Insurance | –  1.28 (0.62-2.65) 0.5062  0.67 (0.34-1.30) 0.2357  0.38 (0.14-1.01) 0.0522  1.27 (0.68-2.36) 0.4577  1.11 (0.40-3.12) 0.8413 | –  **2.76 (1.65-4.63) 0.0001**  **2.12 (1.40-3.23) 0.0004**  **2.35 (1.45-3.80) 0.0006**  **0.45 (0.25-0.81) 0.0078**  0.30 (0.08-1.11) 0.0704 | –  **4.48 (2.32-8.66) <.0001**  **2.97 (1.70-5.20) 0.0001**  **5.74 (3.24-10.18) <.0001**  **0.30 (0.13-0.70) 0.005**  1.10 (0.29-4.22) 0.886 |  |
| Primary Eczema Provider Type  Specialist (ref)  Non-specialist  No Provider | –  1.03 (0.56-1.89) 0.9275  1.27 (0.68-2.36) 0.4577 | –  **2.58 (1.78-3.74) <.0001**  **0.45 (0.25-0.81) 0.0078** | –  1.45 (0.90-2.34) 0.123  **0.30 (0.13-0.70) 0.005** | – |

Supplemental Table 2: Multiple Logistic Regression Model

| **HCP referred to mental health resources** | | | |
| --- | --- | --- | --- |
| **Predictors** | Yes  **OR (95% CI) P-value** | **Predictors** | Yes  **OR (95% CI) P-value** |
| Age  0-17 (ref)  18-34  35-64  65+ | –  0.82 (0.55-1.20)  0.2983  **0.28 (0.18-0.45)**  **<.0001**  **0.09 (0.04-0.22)**  **<.0001** | RUCA  Urban (ref)  Large Rural City/Town  Small/Isolated Rural Town | –  0.83 (0.67-1.04)  0.1148  **2.19 (1.84-2.60)**  **<.0001** |
| Gender  Female (ref)  Male  Other | –  **2.58 (1.90-3.49)**  **<.0001**  0.43 (0.12-1.59)  0.2061 | Education  Advanced (ref)  Limited  Average | –  **4.74 (3.11-7.24)**  **<.0001**  **2.48 (1.79-3.42)**  **<.0001** |
| Race  White (ref)  Asian or Asian American  Black or African American    Multiracial/Other | –  **0.59 (0.35-1.00)**  **0.0493**  1.23 (0.77-1.98)  0.392  0.85 (0.56-1.30)  0.4622 | Insurance Type  Employer-sponsored (ref)  Medicaid or State  Assistance  Medicare/Tricare/VA  Purchased Policy    Other Insurance    No Insurance | –  **3.07 (1.96-4.81)**  **<.0001**  **2.66 (1.81-3.92)**  **<.0001**  **2.91 (1.93-4.39)**  **<.0001**  1.33 (0.57-3.11)  0.5043  0.76 (0.27-2.19)  0.6161 |
| Ethnicity  Non-Hispanic (ref)  Hispanic | –  **2.75 (1.99-3.81)**  **<.0001** | Primary Eczema Provider Type  Specialist (ref)  Non-specialist  No Provider | –  **1.80 (1.30-2.48)**  **0.0003**  **0.28 (0.15-0.53)**  **<.0001** |
| Income  Middle(ref)  Low  High | –  0.85 (0.62-1.16)  0.3053  **0.36 (0.20-0.62)**  **0.0003** |  |  |
